# Digital Occlusal Contact Area Indicates Masticatory Performance for Normal Occlusion, but Not for Malocclusion

**DOI:** 10.1111/joor.14002

**Published:** 2025-05-04

**Authors:** Paulina Vortkamp, Stefan Rues, Sven Räther, Lydia Eberhard, Alexander Rößler, Frederic Bouffleur, Reinald Kühle, Franz Sebastian Schwindling, Peter Rammelsberg, Christoph J. Roser, Christopher Herpel

**Affiliations:** ^1^ Department of Prosthodontics University of Heidelberg Heidelberg Germany; ^2^ Department of Oral and Maxillofacial Surgery University of Heidelberg Heidelberg Germany; ^3^ Department of Prosthetic Dentistry Medical University Innsbruck Innsbruck Austria; ^4^ Department of Orthodontics University of Heidelberg Heidelberg Germany

**Keywords:** CAD/CAM, masticatory performance, occlusal contact area, occlusal rehabilitation

## Abstract

**Objectives:**

Since 1949, occlusal contact area (OCA) has been recognised as an indicator of masticatory performance (MP), but it required individual and laborious analog measurement. Today, a digital workflow could provide automatic quantification.

**Objective:**

To find optimal parameters for a digital evaluation in terms of maximising correlation between OCA and MP.

**Methods:**

MP was measured clinically by comminution of standardised test food by 41 participants (mean age = 29, *n* female = 28), including 18 patients with dentofacial deformities and 23 healthy subjects with normal occlusion. OCA was measured in the laboratory. After impression taking, gypsum casts were digitised (D2000, 3shape) and aligned in maximum intercuspidation (Geomagic Design X2022, 3D Systems). The maxilla was enlarged with surface offsets of 100, 150, 200, 250, 300, 350, 400, 800 and 2000 μm to simulate different interocclusal distances. OCA was identified at the mandible surface by intersection with the respective enlarged maxilla scan (3D surface area). OCA projection onto the occlusal plane (2D area) was also computed, resulting in a total of 18 different OCA evaluations per patient.

**Results:**

MP was lower in patients with malocclusion than in individuals with normal occlusion (X50 mean 5.35 vs. 4.62). The 18 mean OCAs ranged from 11 to 852 mm^2^. In subjects with normal occlusion, strong correlations were shown for interocclusal distances between 150 and 300 μm combined with 3D projection (*r* < −0.7). For patients with malocclusion, no significant correlations were identified.

**Conclusions:**

OCA appears a suitable indicator of MP in individuals with normal occlusion, but not in patients with malocclusion, where other factors seem more determinant.

## Graphical abstract

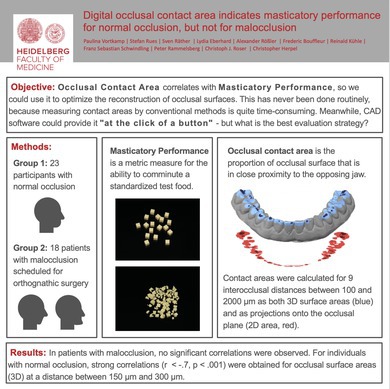

## 1 Introduction

Some of the variability in chewing performance can be explained by variables, such as gender, body size and jaw morphology [1]. A substantial part of the variation, however, is determined by the occlusion [2]. In this regard, the mere number of teeth, tooth contacts or the overall size of the occlusal surfaces proved of less importance. Instead, it is a larger *occlusal contact area* that facilitates the comminution of food [1, 3, 4]. The occlusal contact area (OCA) is the proportion of occlusal surface that comes in close proximity to the opposing jaw during maximum intercuspidation. How close this relationship must be for an occlusal area to be considered part of the OCA has not yet been uniformly established.

In the classic method, as also used by Yurkstat and Manly over 70 years ago, a test person bites with maximum force on a warmed wax plate of 1 mm thickness. The plate is then transilluminated and the transmitted light is measured on a photographic film [5] or using a photoelectric cell [2, 6]. Later, digital image processing techniques [1], pressure‐sensitive films (Dental Prescale Occluzer, Fuji Film) [3, 7, 8] or silicone bite registration materials [9] were applied. Obviously, the size of the contact area calculated via these methods was influenced by the thickness, opacity and hardness of the wax plate used [2] respectively by the characteristics of the pressure film or registration material. As a result, different methods led to different values for the observed contact area. In addition to this variability, the conventional techniques were always associated with considerable experimental and instrumental work, which is why the determination of the occlusal contact area could not be carried out routinely.

This is changing with the advance of digital dentistry, bringing new attention to the analysis of the occlusal contact area. Dental design software could enable automatic, software‐based quantification (‘at the click of a button’) in the sense of a built‐in analysis function, without additional registrations or measurements. The basic approach can be summarised as follows: (i) Digital dental casts of both jaws are generated. (ii) The models are digitally aligned in maximum intercuspidation to each other. (iii) The contact area is the proportion of the occlusal surface of one model that is within a certain distance to the surface of the other model [10]. Obviously, this ‘certain distance’ can be chosen arbitrarily and will determine the size of the resulting contact area [3].

To date, there has been a lack of studies that have determined occlusal contact areas in this digital manner and investigated their relationship with masticatory performance.

Furthermore, it will make a difference whether contact areas are determined in a three‐dimensional space or, as with conventional methods, from a two‐dimensional projection perpendicular to the masticatory plane. Based on these considerations, the present study will compare different evaluation strategies for the digital determination of the contact area that vary in terms of interocclusal distance and geometric projection. The measurement of masticatory performance will be carried out using the established digital sieving method to ensure comparability with previous studies [11]. To increase the representativeness of the results, in addition to a group of subjects with normal occlusion, the study will also include patients with malocclusion requiring surgical treatment, since increasing contact area and the associated improvement in masticatory performance is a primary treatment goal for these patients [12].

Thus, this study included two populations: Individuals with normal occlusion and patients with malocclusion requiring surgical treatment. The intervention involved the measurement of masticatory performance and the digital determination of occlusal contact area. Comparisons were made between different evaluation strategies for occlusal contact area that differed in terms of interocclusal distance and geometric projection. The outcome and aim of this study were to determine, for both groups, the optimum evaluation strategies that correlated most strongly with masticatory performance.

## 2 Methods

### 2.1 Study Design

An exploratory cross‐sectional study was initiated at Heidelberg University Hospital after approval had been obtained from the local Ethics Committee. Due to the exploratory nature of the study, a number of 40 individuals was determined a priori. Twenty patients, each with normal occlusion and malocclusion, were to be included. For the group with normal occlusion, a convenience sample of students from the medical faculty of the University of Heidelberg was to be included. Exclusion criteria were dental or skeletal malocclusions. Inclusion criteria were a minimum age of 18 years and the presence of bilateral Angle Class I occlusion (normal occlusion). Normal occlusion was assumed if each tooth had at least one occlusal antagonistic contact. Furthermore, any deviation from Angle Class I dentition in the area of the canines and first molars had to be within a maximum discrepancy of half a premolar width.

For the group with malocclusion, consecutive patients were included for whom orthognathic surgery was planned at Heidelberg University Hospital and who were referred to the Department of Oral and Maxillofacial Surgery for this purpose. Patients were also included if they were scheduled for orthognathic surgery following an internal referral from the Department of Orthodontics. Inclusion criteria were a minimum age of 18 years and the presence of skeletal malocclusion indicated for bimaxillary osteotomy. Patients were excluded if orthodontic treatment had already begun, that is, if they were wearing braces.

After written informed consent was obtained, patients were called in for a separate appointment. At this visit, participants were required to masticate a silicone test food in order to calculate masticatory performance. In addition, impressions of the upper and lower jaws were taken to calculate the occlusal contact area. To further characterise the sample, all participants underwent an examination according to the diagnostic criteria for temporomandibular disorders (DC/TMD) to describe the presence of pain‐related and non‐pain‐related functional limitations in the sample.

### 2.2 Occlusal Contact Area

**FIGURE 1:**
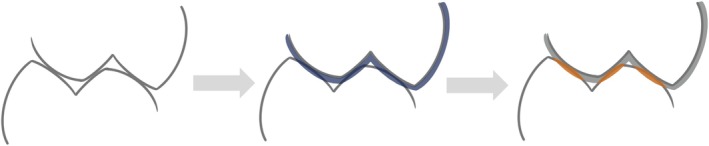
Principle for the digital determination of the occlusal contact area: As soon as digital models of both jaws are positioned in maximum intercuspation (left), the model of the maxilla is enlarged by a certain offset value (center) and the contact area is calculated from the size of the mandibular occlusal surfaces intersecting the enlarged upper maxillary model (right).

Figure 1 illustrates the principle for the digital OCA determination used in this study. (i) For each patient, 3D surfaces of digitised models of maxilla and mandible were aligned in maximum intercuspation. (ii) Enlargement of the maxilla by a given surface offset parameter. (iii) After intersection of the lower jaw scan with the enlarged maxilla, OCA was defined by trimming the lower jaw surface using the intersection lines.

**FIGURE 2:**
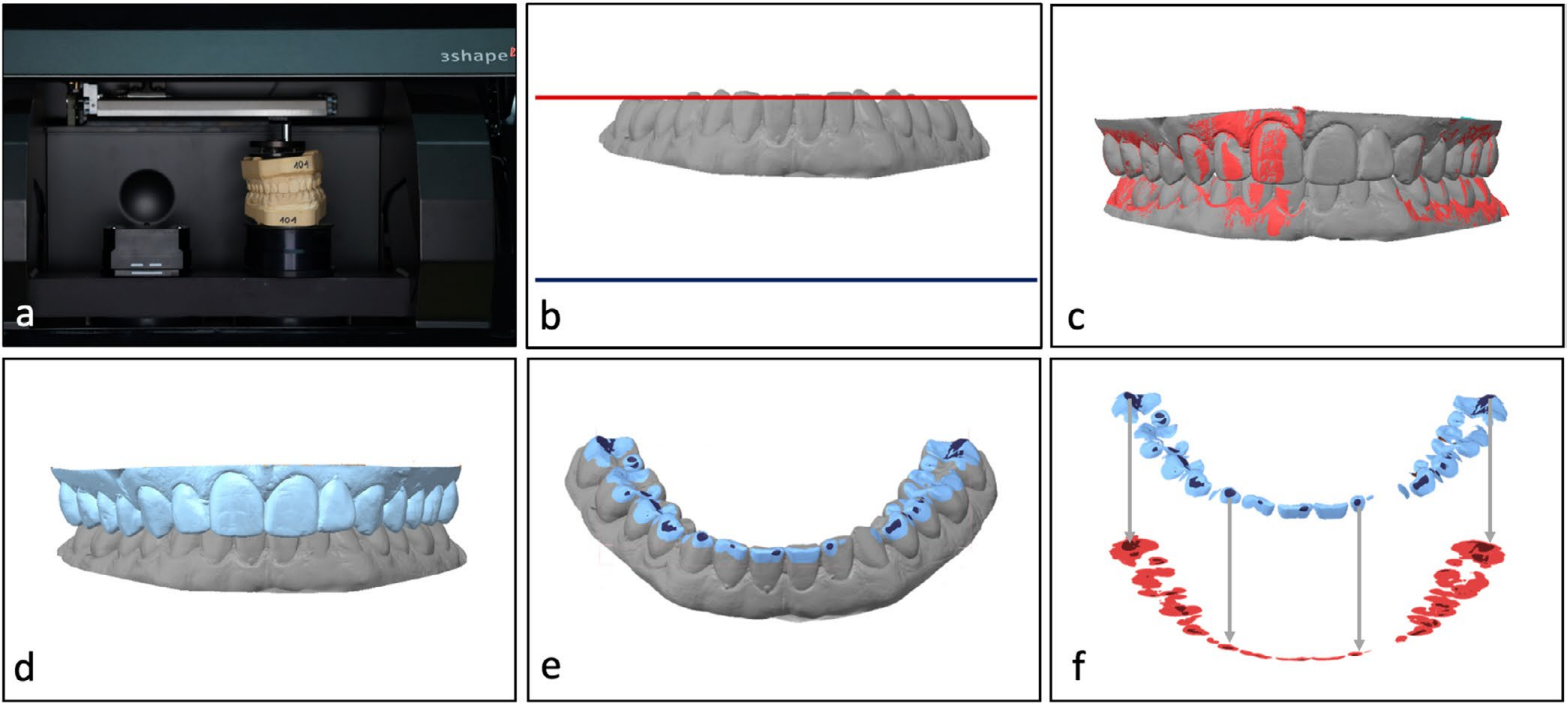
Workflow for the different strategies to evaluate occlusal contact area (OCA): (a) After conventional impression taking, gypsum casts were digitised (D2000, 3shape) and (b, c) aligned in maximum intercuspidation using CAD‐software (Geomagic Design X2022, 3D Systems). (d) The maxilla was enlarged with 9 different offsets ranging from 100 to 2000 μm, and (e) OCA was calculated as the respective intersection of both occlusal surfaces (3D surface area). In this example, the contact area is shown for distances of 200 (dark blue) and 800 μm (light blue). (f) Each surface area was also projected onto the occlusal plane (2D area, light red/dark red). This corresponded to the conventional method of determining OCA via transillumination of a wax record.

The detailed study‐specific laboratory procedure is shown in Figure 2. The study was carried out in a hybrid workflow: (a) During appointments, conventional polyether impressions (Impregum Penta Soft, 3M) of maxilla and mandible were taken as well as a bite registration in habitual maximum intercuspation (Futar D fast, Kettenbach). Based on the impressions, gypsum casts (Fuji Rock, GC Europe) were fabricated. The individual models were then digitised with an optical desktop scanner (D2000, Convince 2016 software, 3shape). The two plaster models were then fixed to each other in maximum intercuspidation. The bite registration was used to identify correct positioning but was not used in the end for the fixation itself in order to prevent any resulting gaps between the occlusal surfaces. Finally, a third scan showing the buccal surfaces of the two combined models was taken. (b) The digital models of both jaws were imported into a CAD software (Geomagic Design X 2022, 3D Systems). First, the mandibular scan was oriented such that the occlusal plane (defined by the contact point of the incisal edges of teeth 31 and 41 and the distobuccal cusps of teeth 36 and 46) was oriented in parallel to the horizontal xy‐plane. (c) Using the buccal scan of the combined models, the maxillary surface was then aligned with the mandibular surface. In detail, the best‐fit method was first applied to the mandibular scan and the combined scan and subsequently to the combined scan and the maxillary scan. The meshes of the separate jaw models are shown here in grey, and the mesh of the buccal scan in red. (d) The maxilla was then gradually enlarged (blue surface) by the offsets of 100, 150, 200, 250, 300, 350, 400, 800 and 2000 μm. (e) Hereby, an intersection between the two occlusal surfaces was generated by each offset value, and the intersection areas at the lower jaw defined the respective occlusal contact area. The figure shows exemplary intersections for offset values of 200 (dark blue) and 800 μm (light blue). (f) The summed surface area of the resulting ‘snippets’ corresponded to the OCA as a 3D surface area (blue). In addition, 3D occlusal contact surface areas were projected into the xy‐plane in order to form a planar image of the contact areas (2D occlusal contact area, illustrated in red colour), corresponding to the conventional method of transilluminating a wax record. This procedure was carried out for each of the 9 offsets. As a result, 18 different surface areas were calculated for the OCA for each participant.

### 2.3 Masticatory Performance

**FIGURE 3:**
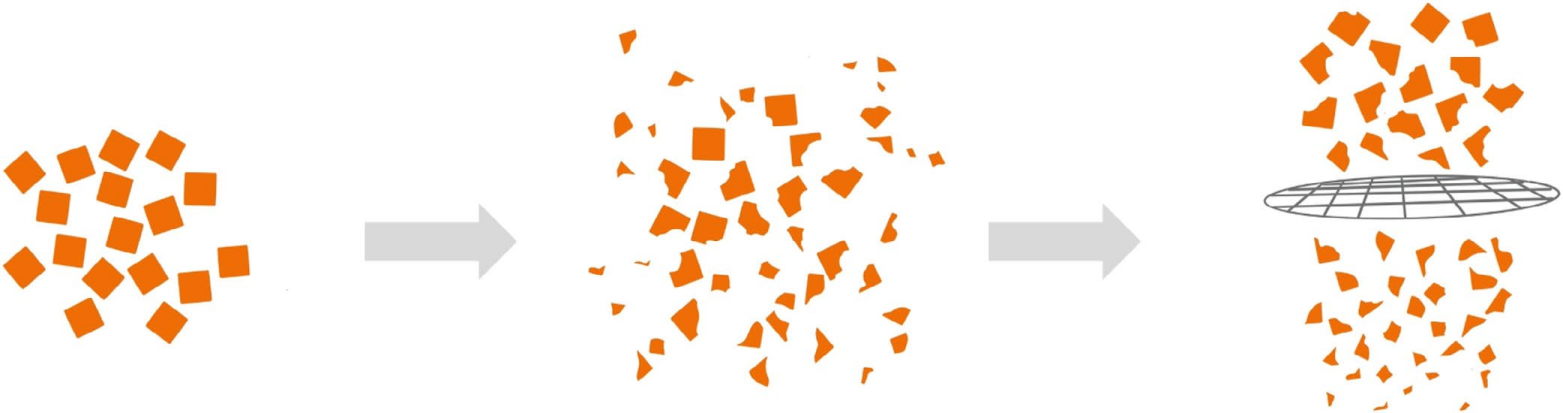
Principle of the sieving method for measuring chewing performance. A test subject chews a standardised test food (left) for a defined number of chewing cycles, the resulting particles are collected (middle). The chewing performance is defined as the mesh size of a theoretical sieve (right) through which 50% of the chewing sample would fall (X50 value). In our study, the value was not determined by actually sieving, but by image processing software.

Masticatory performance was measured using the established digital sieving method (Figure 3) according to the procedure developed by our working group [11]. Participants are instructed to chew a standardised test food for a defined number of chewing strokes and then to spit it out. The digital sieving method replicates the classic procedure of the analog sieving method by means of image analysis. Instead of sequential sieving, the chewed particles are spread out on a flatbed scanner and separated from each other. Based on the particle size distributions, regression analysis is used to calculate the mesh size of a theoretical sieve through which 50% of the test food would fall. This ‘X50 value’ is the resulting metric measure of ‘masticatory performance’ (Fiji [13]/MATLAB, Mathworks). A lower value corresponds to a higher masticatory performance.

Each participant received a standardised, cubic test food made from impression silicone (Optsosil Comfort Putty, Kulzer). Masticatory performance testing was carried out twice, with only the second evaluation being included in the analysis. This was in line with the methodology used in the relevant literature and is intended to allow the participants to get accustomed to the test food in the first round and to meet the requirement of a ‘natural, unforced chewing’ in the second round. Each portion of test food (17 cubes, edge length 5 mm) was weighed before chewing. Each portion was chewed for 15 chewing strokes, with the examiner counting and giving start and stop signals. After chewing, the particles were collected in a filter and the participant rinsed the mouth with water to capture all the test food as far as possible. The particles were then disinfected with 70% alcohol and dried. Before the analysis, the entire test portion was weighed again so that the actual weight of the collected chewing sample could be taken into account when calculating chewing performance.

### 2.4 Statistical Analysis

Statistical analyses explored the association between masticatory performance (MP) and OCA among two groups of patients with malocclusion and normal occlusion. Descriptive statistics, including means, medians, quartiles, and range, were computed for MP and OCA. Box plots were generated to visually display these distributions. The relationship between MP and the 18 different evaluation strategies for OCA was examined using Pearson correlation coefficients, and line diagrams were used to illustrate these correlations. For both the total sample and the subgroups, the OCA evaluation strategy that showed the strongest correlation with MP was to be identified, and scatter plots were created to further investigate the relationships. Differences in MP between the two groups were assessed using the Mann–Whitney *U*‐test. All analyses were conducted using SPSS Version 25 with a significance threshold set at a *p* value of less than 0.05.

## 3 Results

### 3.1 Participants

**FIGURE 4:**
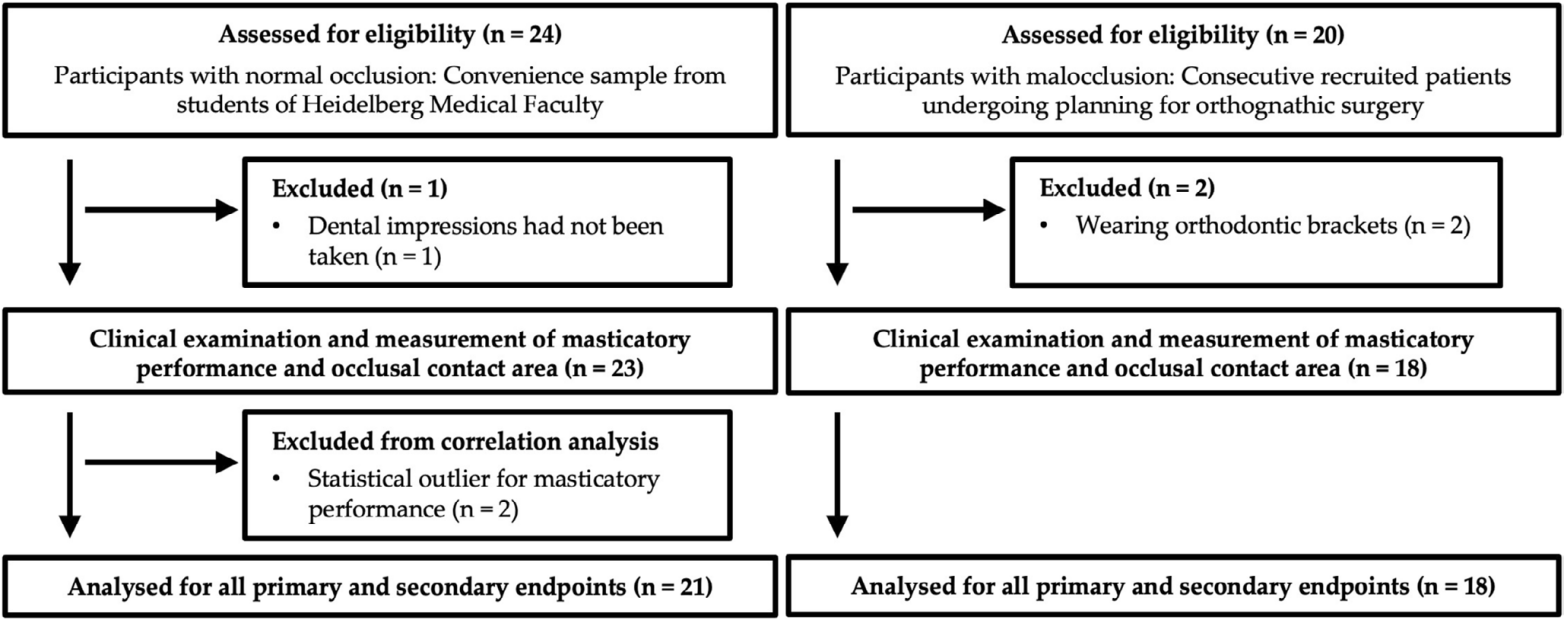
Study flow chart of participants.

Participants were recruited for this study from October 2021 to December 2022, as depicted in the study flow chart (Figure 4). In the normal occlusion group, 24 participants were initially screened and could be included after normal occlusion was confirmed. For one patient, determination of the occlusal contact area was not possible since dental impressions had not been taken. For the malocclusion group, 20 patients were screened; of these, two were excluded because of existing orthodontic brackets. The remaining participants underwent all evaluations for MP and OCA. After descriptive analysis, two participants from the normal occlusion group were identified as statistical outliers and excluded from correlation analysis due to their exceptionally low MP/X50 value, exceeding 2 standard deviations from the mean.

The demographic and clinical characteristics of both groups are presented in Table 1, showing similar distributions in age and gender. In the malocclusion group, Angle Class II was the predominant classification (50%). Subjective masticatory impairment, defined as a mean score greater than 1 on the Jaw Functional Limitation Scale questionnaire mastication subscale, was reported by 2 (9%) participants in the normal occlusion group and 10 (56%) in the malocclusion group. Additionally, 4 participants in the normal occlusion group and 9 in the malocclusion group were diagnosed with myofascial orofacial pain, fulfilling the relevant diagnostic criteria for temporomandibular disorders (DC/TMD).

**TABLE 1:** Characteristics of the participants with normal‐occlusion and those with malocclusion.

Parameter	Normal occlusion (*n* = 23) median (range) or count (%)	Malocclusion (*n* = 18) median (range) or count (%)
Age	25 (23–33)	30 (18 to 51)
Gender		
Female	18 (78%)	10 (56%)
Male	5 (22%)	8 (44%)
Angle class (*n*)		
I	23 (100%)	4 (22%)
II	—	9 (50%)
III	—	5 (28%)
Open bite	0	9 (50%)
Overjet (mm)	2 (1–5)	2 (−5 to 12)
Overbite (mm)	2 (1–4)	0.5 (−5 to 7)
Subjective masticatory impairment^a^	0 (0–2.7)	1.6 (0 to 7)
GCPS‐grading for painful TMD^b^		
0	19 (83%)	9 (50%)
I	4 (17%)	7 (39%)
II	—	2 (11%)

^a^ Based on the Jaw Functional Limitation Masticatory Subscale (JFLS, 20‐item questionnaire).

^b^ GCPS: graded chronic pain scale, relating to myofascial orofacial pain according to the ‘diagnostic criteria of temporomandibular disorders’ (DC/TMD).

### 3.2 Masticatory Performance

The two measurements of chewing performance yielded a high retest reliability with Pearson's *r* = 0.862 (normal occlusion) and 0.842 (malocclusion), respectively *p* < 0.001. The second measurement that was included in the analysis provided a mean masticatory performance of X50 = 4.58 (SD: 0.37) in the normal occlusion group. The patients with malocclusion showed a significantly lower masticatory performance of X50 = 5.35 (SD: 0.43) (Mann–Whitney *U* test, *p* < 0.001, Figure S1).

### 3.3 Occlusal Contact Area

For the occlusal contact area, 18 values were calculated for each participant according to the evaluation strategies varying in interocclusal distance and projection. Figure S2 shows the distribution of the values for the group with normal occlusion under evaluation in 2D projection (light red) and in 3D projection (dark red). Figure S3 illustrates the corresponding occlusal contact areas of the group with malocclusion. As expected, OCA increased with increasing interocclusal distance. At the same time, the contact areas in 2D projection were consistently smaller. Hence, a larger offset resulted in larger contact areas, ranging from 11 mm^2^ (SD 6 mm^2^) at 100 μm and 2D projection to 852 mm^2^ (SD 201 mm^2^) at 2000 μm and 3D projection.

### 3.4 Correlation

**FIGURE 5:**
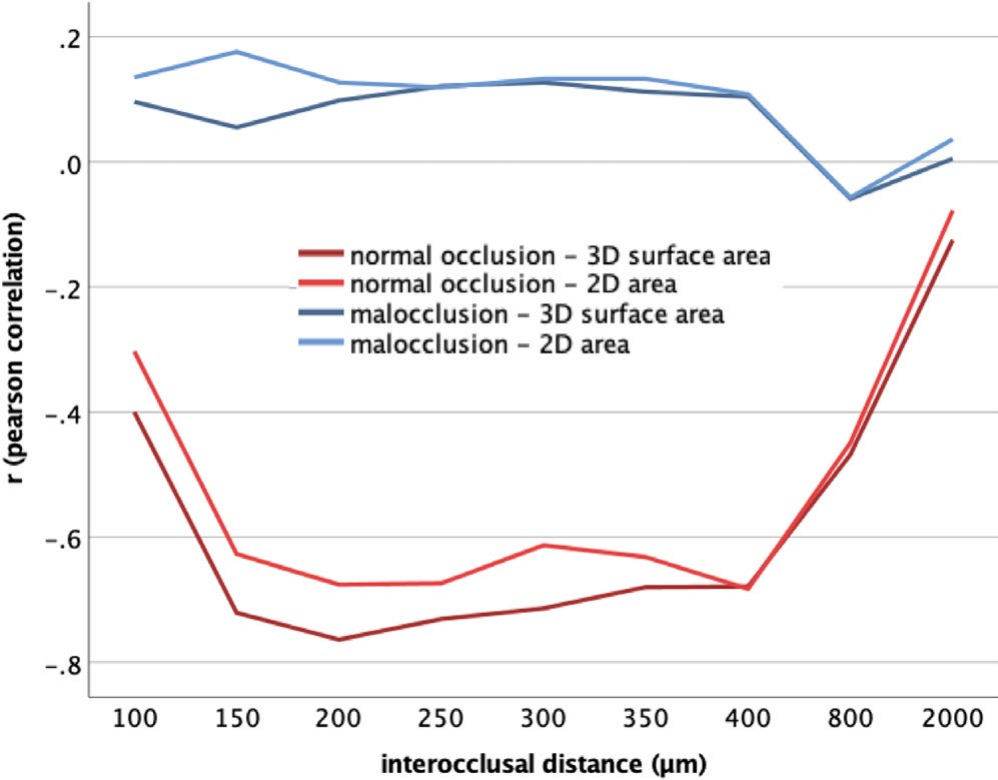
Correlation between masticatory performance and occlusal contact area as a function of interocclusal distance:In patients with malocclusion, no significant correlations were observed (blue lines). For individuals with normal occlusion, strong correlations (*r* < −0.7, *p* < 0.001) were obtained for occlusal surface areas (3D) at a distance between 150 and 300 μm (dark red line).

Figure 5 illustrates the strength of correlations between MP and OCA across all 18 evaluation strategies. Since a higher masticatory performance is expressed by a lower X50 value, corresponding correlations are expected to be negative. In the group of patients with malocclusion, no significant correlations were found. For the participants with normal dentition, however, strong correlations (Pearson's *r* < −0.7) were found for surface areas (3D projection) between 150 and 300 μm offset. Figure S4 visualises the linear relationship between occlusal contact area at 200 μm offset and 3D projection, the evaluation strategy for which the strongest correlation was found (Pearson's *r* = −0.764, *p* < 0.001).

## 4 Discussion

Patients with malocclusion showed lower masticatory performance than subjects with normal occlusion. For the occlusal contact area, 18 different evaluation strategies were investigated by assuming 9 different interocclusal distances between 100 and 2000 μm and evaluating each in 2D (contact area) and 3D projection (contact surface area). In patients with malocclusion, no significant correlations with masticatory performance were observed over the entire interocclusal distances examined. For the participants with normal dentition, the strongest correlations (Pearson's *r* < −0.7, *p* < 0.001) were found for contact surface areas (3D projection) at interocclusal distances between 150 and 300 μm.

In the relevant literature, corresponding correlations with masticatory performance have been described for occlusal contact areas determined by conventional means. Wilding et al. described, using almonds as test food, a significant correlation for intermediate occlusal contact areas (200–450 μm interocclusal distance) and the absence of any correlation for tighter contact areas up to a distance of 200 μm [14, 15]. Owens et al. investigated the relationship between masticatory performance determined using a silicone test food and the size of the occlusal contact areas between 50 and 350 μm. In their results, the strongest correlation was observed for evaluations between 200 and 300 μm [3]. Using an Optosil test sample, Lujan‐Climent et al. measured contact areas in increments of 50 μm between 0 and 500 μm. They reported a fairly constant moderate correlation between 50 and 300 μm of interocclusal distances [16]. Horie et al. used a digital device (Bite Eye BE‐I, Tokyo) to determine the OCA after taking silicone bite registrations. Although distances between 50 and 200 μm were examined, the strength of the correlation with the MP increased up to an interocclusal distance of 160 μm. In this study, MP was measured via colour differences of a colour‐changing chewing gum [17]. In a more recent study, Lee et al. also used the BiteEye device to determine OCA from a silicone registration and the sieving method for masticated peanuts to determine MP. They found the greatest correlation at an interocclusal distance of 149 μm, which was also the largest distance examined in this study [18]. Although the aforementioned studies reported correlations that were partially consistent with our results, the methods used to measure masticatory performance and contact area diverged. The aim of the present investigation was to evaluate contact surfaces using digitised models within CAD software, as such an evaluation would in principle be possible ‘along the way’—and corresponding studies were lacking. If the occlusal contact area were to be provided as standard within CAD software, it could serve as an additional, objective target parameter when occlusal surfaces have to be reconstructed as part of prosthetic interventions.

In the present study, no correlations were found between MP and OCA in patients with malocclusion. In contrast, the study by Owen et al. also included patients with malocclusion and identified significant correlations for a mixed sample [3]. However, these were patients who were scheduled for orthodontic treatment only, with no indication for orthognathic surgery. Overall, the available studies provided only little information on the occlusal characteristics of their participants. However, our results are consistent with the findings of Sasaki et al. who concluded that in patients with more severe malocclusion, MP is less determined by occlusion than by various other factors such as the surrounding soft tissues including the tongue and lips [19]. It can therefore be assumed that the OCA is suitable as an indicator of MP in individuals with normal dentition, but that other factors play a major role in patients with severe malocclusion.

Important limitations of our results are that the evaluation was based on a rather small sample. Furthermore, in addition to OCA, other factors that were not investigated in the present study will also be important, such as the design of the occlusal surface, including cusps and fissures, the individuals body weight, physical constitution and age.

It may also be discussed that in this study, a hybrid procedure was applied, with digitization of plaster models obtained from conventional impressions. Alternatively, direct intraoral scans and a jaw relationship using a buccal scan would have been possible. It has been shown that the determination of OCA using digitised plaster models provides comparable results to the classic prescale method applied to plaster models [10]. However, with direct intraoral scanning, the size of the contact surfaces could have deviated from the corresponding situation on plaster models. However, currently available scanning devices still seem to perform inferior to conventional impressions in terms of the dimensions of a full‐arch image [20].

Finally, the identified correlations describe the strength of the relationship only for a specific test setting and a specific study population (younger subjects with largely intact occlusal surfaces without signs of advanced tooth structure loss). This means that we can hardly make conclusions about the correlations with other test diets, and the generalisability of our results to other populations must be considered with caution. However, the test chosen in the present study using silicone cubes and evaluation using the sieving method is probably the most common and established method in the literature [11]. It also seems reasonable to choose a test material such as silicone (Optosil Putty), as good chewing performance will be of particular clinical interest for relatively hard and tough foods.

## 5 Conclusions

Occlusal contact area correlates with masticatory performance, so it could be used to optimise the reconstruction of occlusal surfaces. This has never been done routinely because measuring contact areas using conventional methods was quite time‐consuming. Meanwhile, CAD software could automatically provide it as a default parameter. Based on the results of this study, the following evaluation strategy can be recommended to obtain the strongest correlation between contact area and masticatory performance: (i) Interocclusal distance from < 150 to < 300 μm. (ii) Evaluation of actual surfaces rather than projections onto the occlusal plane. Notably, this appears to apply only to individuals with unworn, normal occlusion and not to patients with severe malocclusion, where other factors appear to be dominant.

## Author Contributions

**Paulina Vortkamp:** investigation, data curation, writing – original draft. **Stefan Rues:** methodology, software. **Sven Räther:** data curation, writing – review and editing. **Lydia Eberhard:** supervision, methodology. **Alexander Rößler:** investigation, resources. **Frederic Bouffleur:** investigation, resources. **Reinald Kühle:** investigation, resources. **Franz Sebastian Schwindling:** supervision, writing – review and editing. **Peter Rammelsberg:** supervision, writing – review and editing. **Christoph J. Roser:** investigation, resources. **Christopher Herpel:** conceptualization, data curation, formal analysis, visualisation, writing – original draft, writing – review and editing.

## Ethics Statement

Ethical approval for this study was obtained from the Ethics Committee of the Medical Faculty of Heidelberg University (approval number S‐855/2021), and all procedures were conducted in accordance with the Declaration of Helsinki. Informed consent was obtained from all participants prior to their inclusion in the study.

## Conflicts of Interest

The authors declare no conflicts of interest.

## Peer Review

The peer review history for this article is available at https://www.webofscience.com/api/gateway/wos/peer‐review/10.1111/joor.14002.

## Supporting information


**Figure S1.** Patients with malocclusion had a lower masticatory performance (X50 mean 5.35) than individuals with normal occlusion (X50 mean 4.58, *p* < 0.001).
**Figure S2.** Sizes of the occlusal contact surfaces as a function of the interocclusal distances selected from 100 to 2000 μm (group with normal occlusion) in 3D projection (light red) and 3D projection (dark red).
**Figure S3.** Sizes of the occlusal contact surfaces as a function of the interocclusal distances selected from 100 to 2000 μm (group with malocclusion) in 3D projection (light blue) and 3D projection (dark blue).
**Figure S4.** Scatterplot showing the correlation between masticatory performance and occlusal contact area as 3D surface area at 200 μm interocclusal distance in the group with normal dentition (Pearson’s *r* = −0.764, *p* < 0.001).

## Data Availability

The data that support the findings of this study are available from the corresponding author upon reasonable request.
