# Supplementary material for: Digital Occlusal Contact Area Indicates Masticatory Performance for Normal Occlusion, but Not for Malocclusion
Source: J Oral Rehabil. 2025 May 4;52(9):1369–76. doi: 10.1111/joor.14002 (PMC12408952; doi:10.1111/joor.14002)
Supplement: Supplementary file 1 — Figure S1. Patients with malocclusion had a lower masticatory performance (X50 mean 5.35) than individuals with normal occlusion (X50 mean 4.58, p < 0.001). Figure S2. Sizes of the occlusal contact surfaces as a function of the interocclusal distances selected from 100 to 2000 μm (group with normal occlusion) in 3D projection (light red) and 3D projection (dark red). Figure S3. Sizes of the occlusal contact surfaces as a function of the interocclusal distances selected from 100 to 2000 μm (group with malocclusion) in 3D projection (light blue) and 3D projection (dark blue). Figure S4. Scatterplot showing the correlation between masticatory performance and occlusal contact area as 3D surface area at 200 μm interocclusal distance in the group with normal dentition (Pearson’s r = −0.764, p < 0.001). [file JOOR-52-1369-s001.docx]

**Digital occlusal contact area indicates masticatory performance for normal occlusion, but not for malocclusion – Supplemental material**


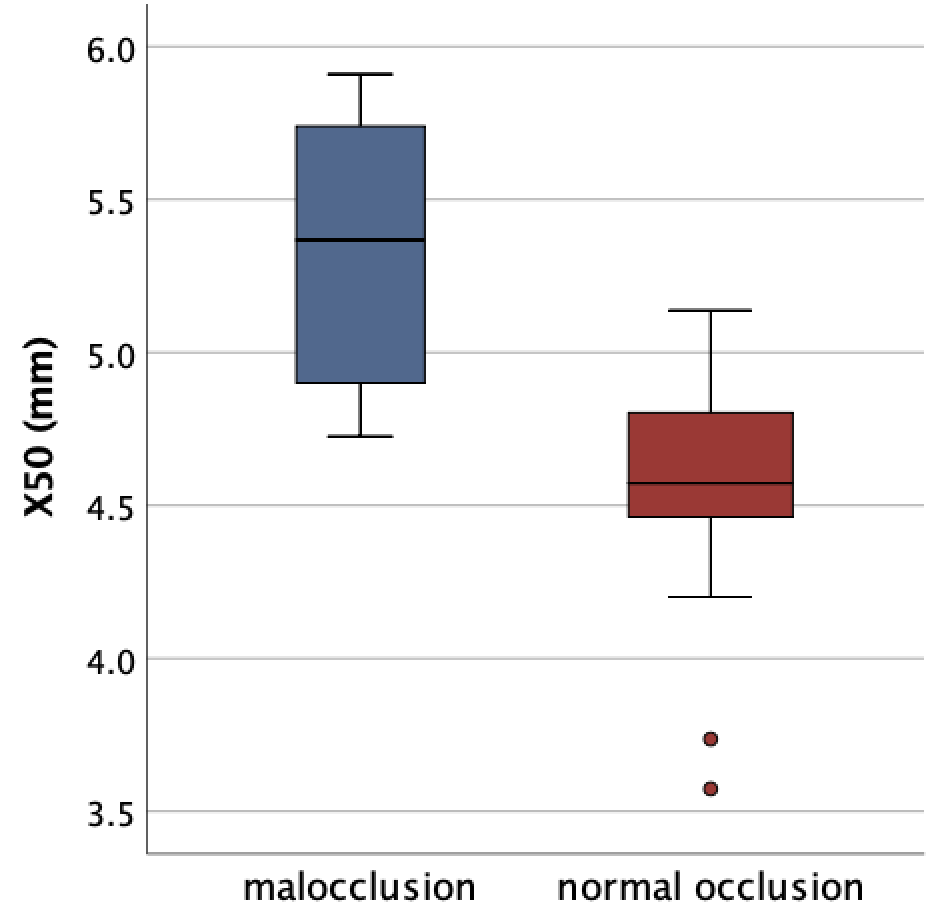


Figure S1: Patients with malocclusion had a lower masticatory performance (X50 mean 5.35) than individuals with normal occlusion (X50 mean 4.58, p < .001). Boxes represent the interquartile range (IQR) with the median line inside; whiskers extend to the minimum and maximum values within 1.5 x IQR, while points beyond are outliers.


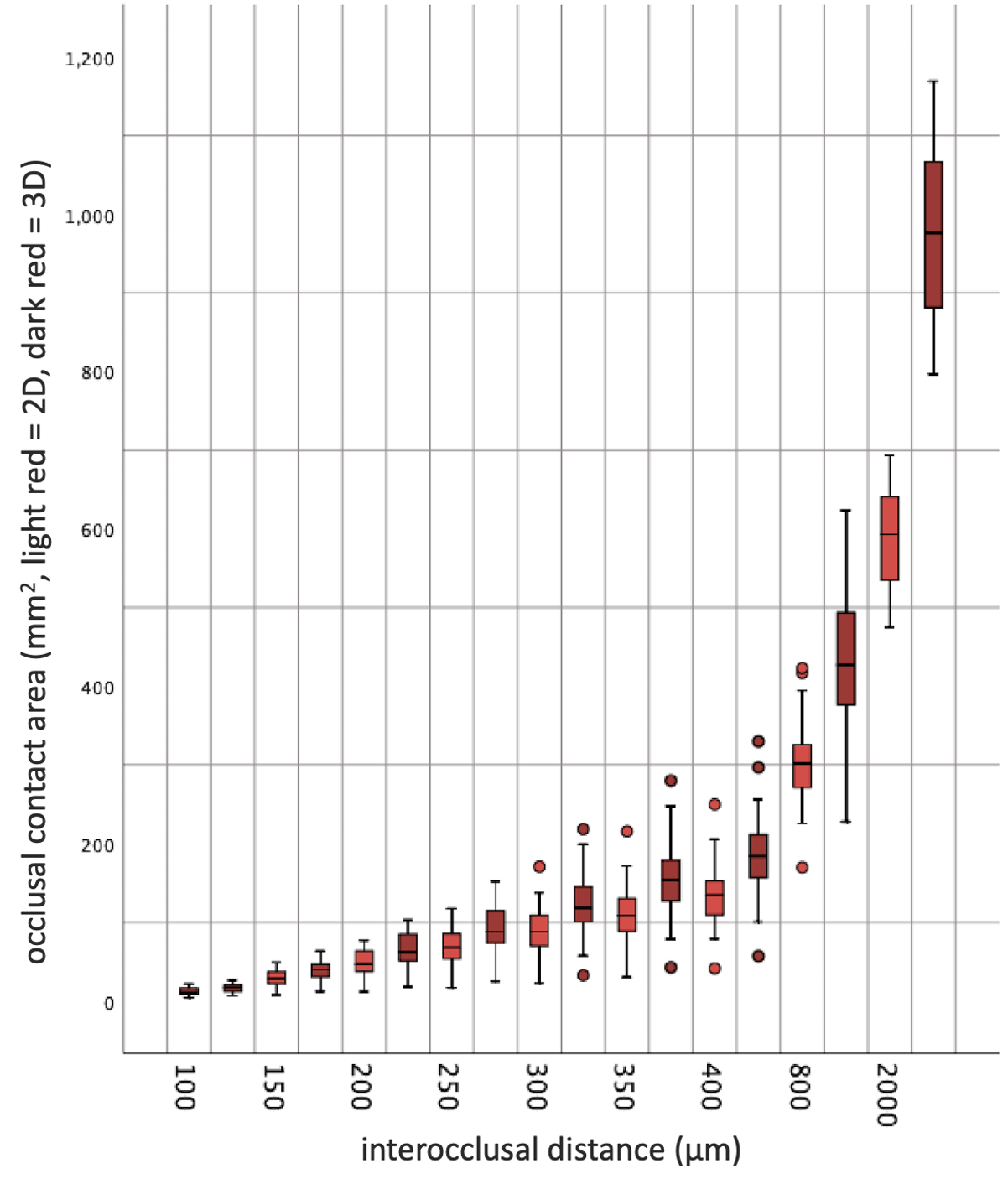


Figure S2: Sizes of the occlusal contact surfaces as a function of the interocclusal distances selected from 100 µm to 2000 µm (group with normal occlusion) in 3D projection (light red) and 3D projection (dark red). Boxes represent the interquartile range (IQR) with the median line inside; whiskers extend to the minimum and maximum values within 1.5 x IQR, while points beyond are outliers.


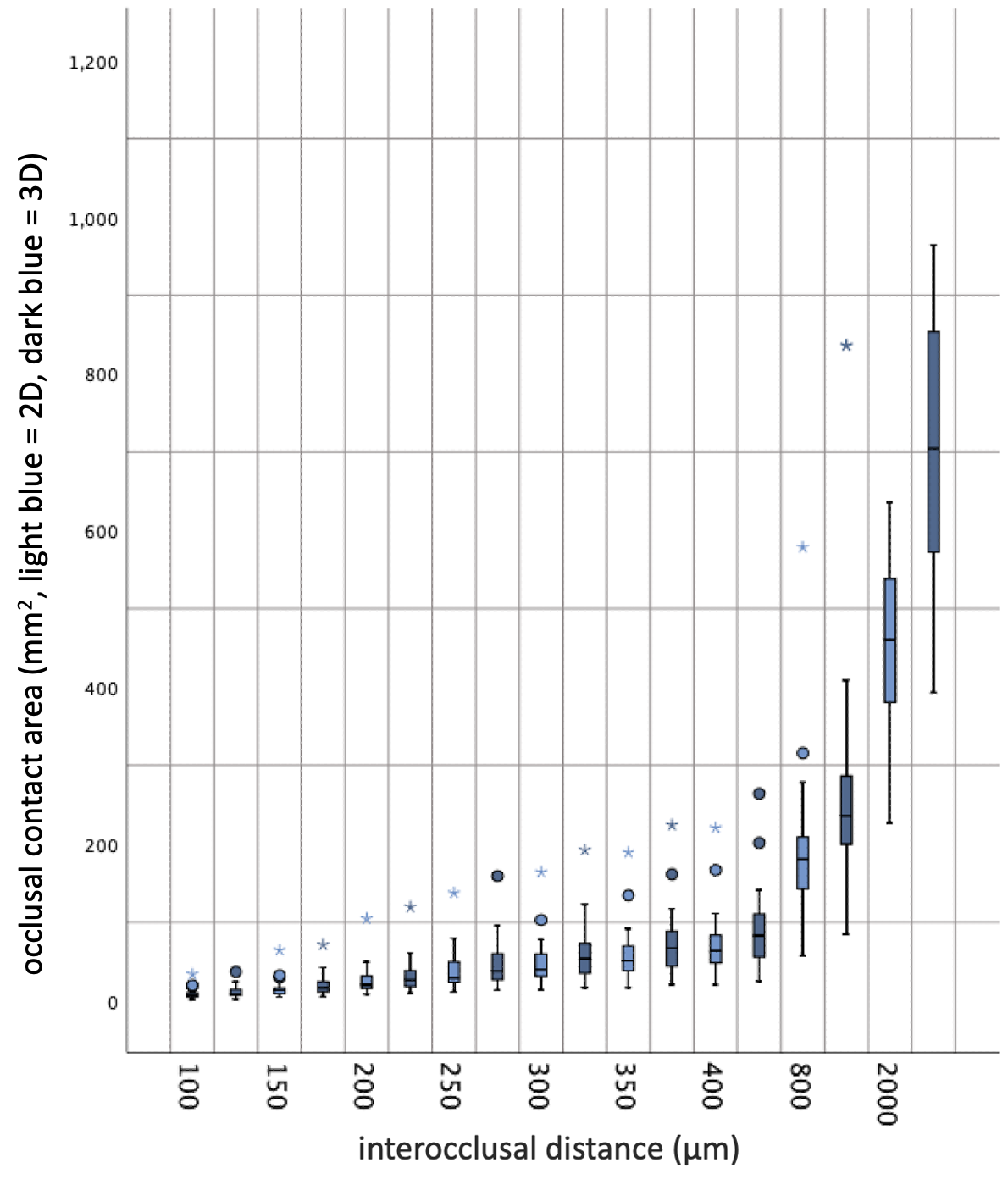


Figure S3: Sizes of the occlusal contact surfaces as a function of the interocclusal distances selected from 100 µm to 2000 µm (group with malocclusion) in 3D projection (light blue) and 3D projection (dark blue). Boxes represent the interquartile range (IQR) with the median line inside; whiskers extend to the minimum and maximum values within 1.5 x IQR, while points and asterisks beyond are outliers.

*
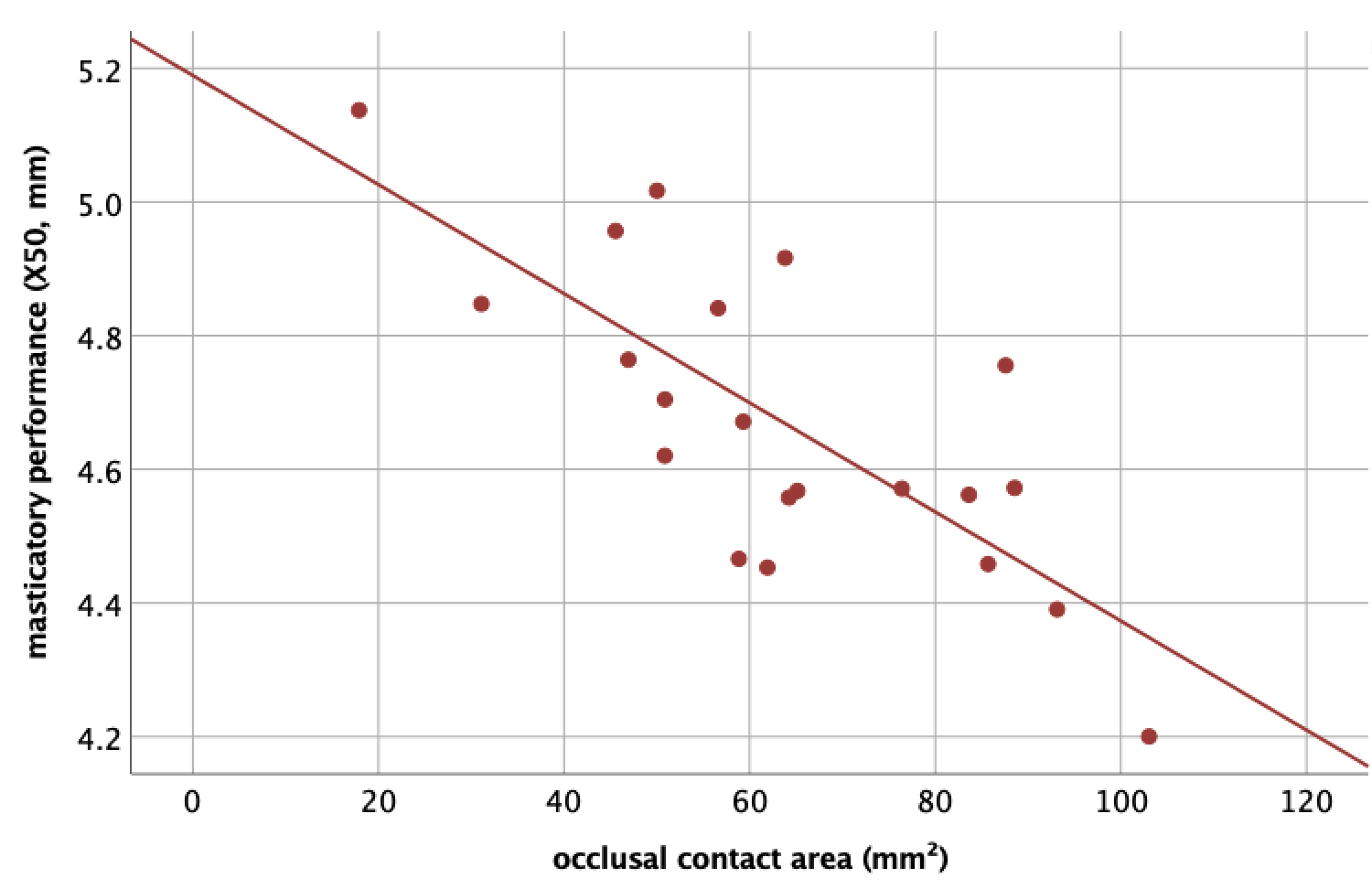
*

Figure S4: Scatterplot showing the correlation between masticatory performance and occlusal contact area as 3D surface area at 200 µm interocclusal distance in the group with normal dentition (pearson's r = -.764, p < .001).
